# Supplementary material for: Soil microbiome analysis reveals effects of periodic waterlogging stress on sugarcane growth
Source: PLoS One. 2023 Nov 2;18(11):e0293834. doi: 10.1371/journal.pone.0293834 (PMC10621937; doi:10.1371/journal.pone.0293834)
Supplement: S1 Fig — Co-occurrence networks including (A) N-BAF, (B) N-BAT, (C) W-BAF, and (D) W-BAT. Node color represents domain (pink: archaea, blue: bacteria, and yellow: fungi). Size of the node is the average relative abundance based on three growth stages (TP, GP, and RP). Blue and red edges mean positive and negative correlations, respectively. Intensity of edge color represents a range of correlations. (DOCX) [file pone.0293834.s001.docx]

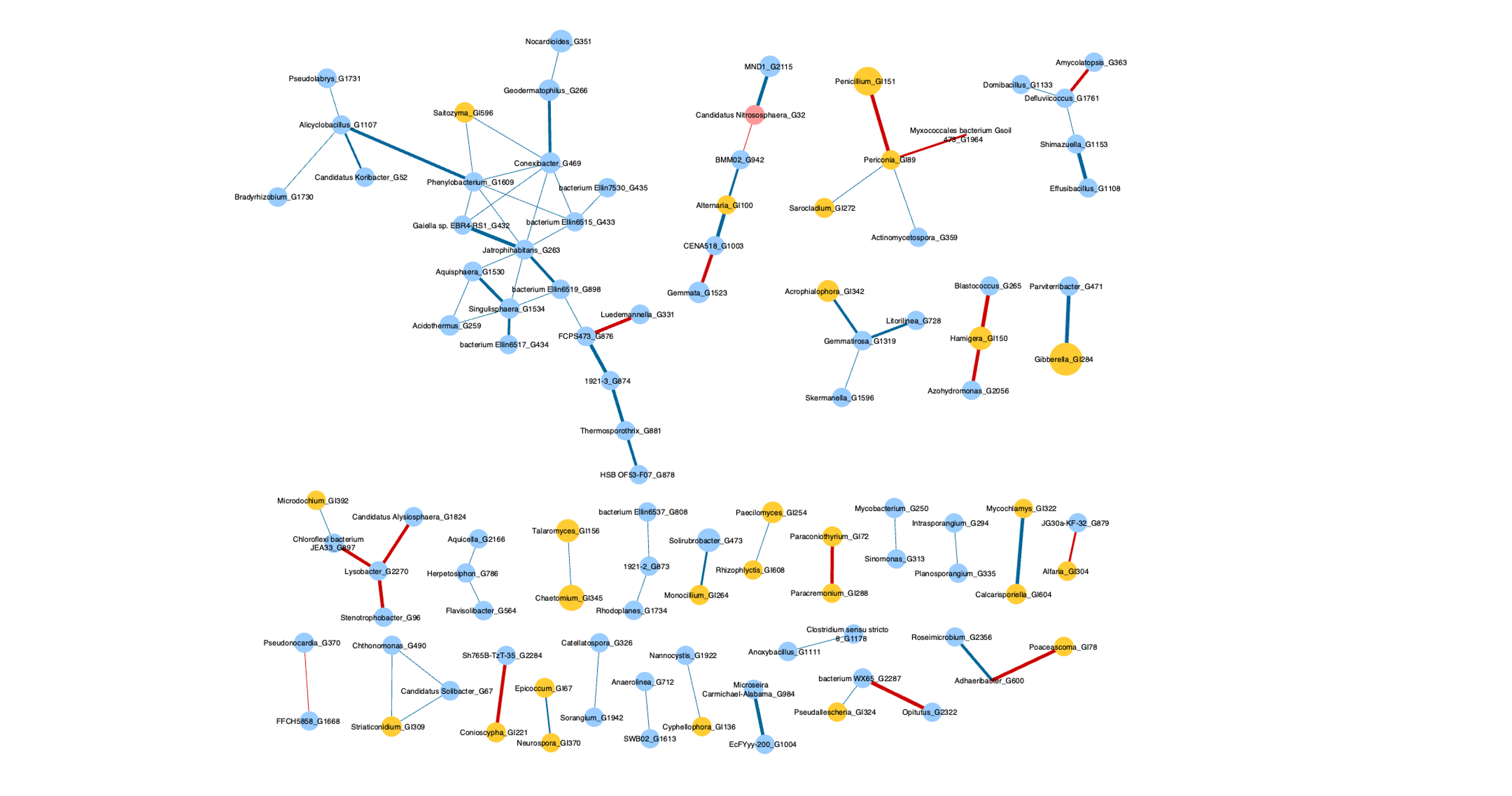


**A**


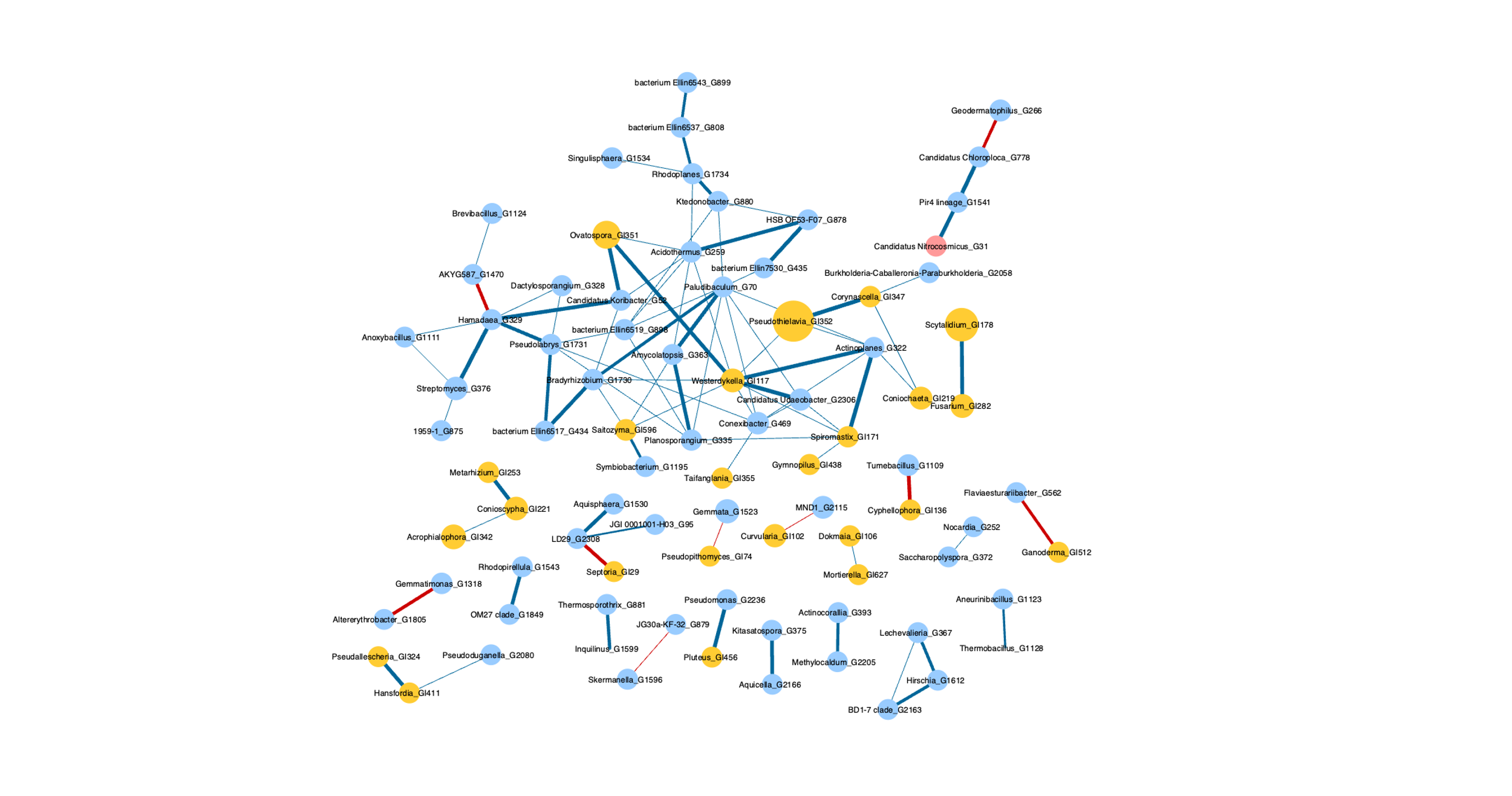


**B**


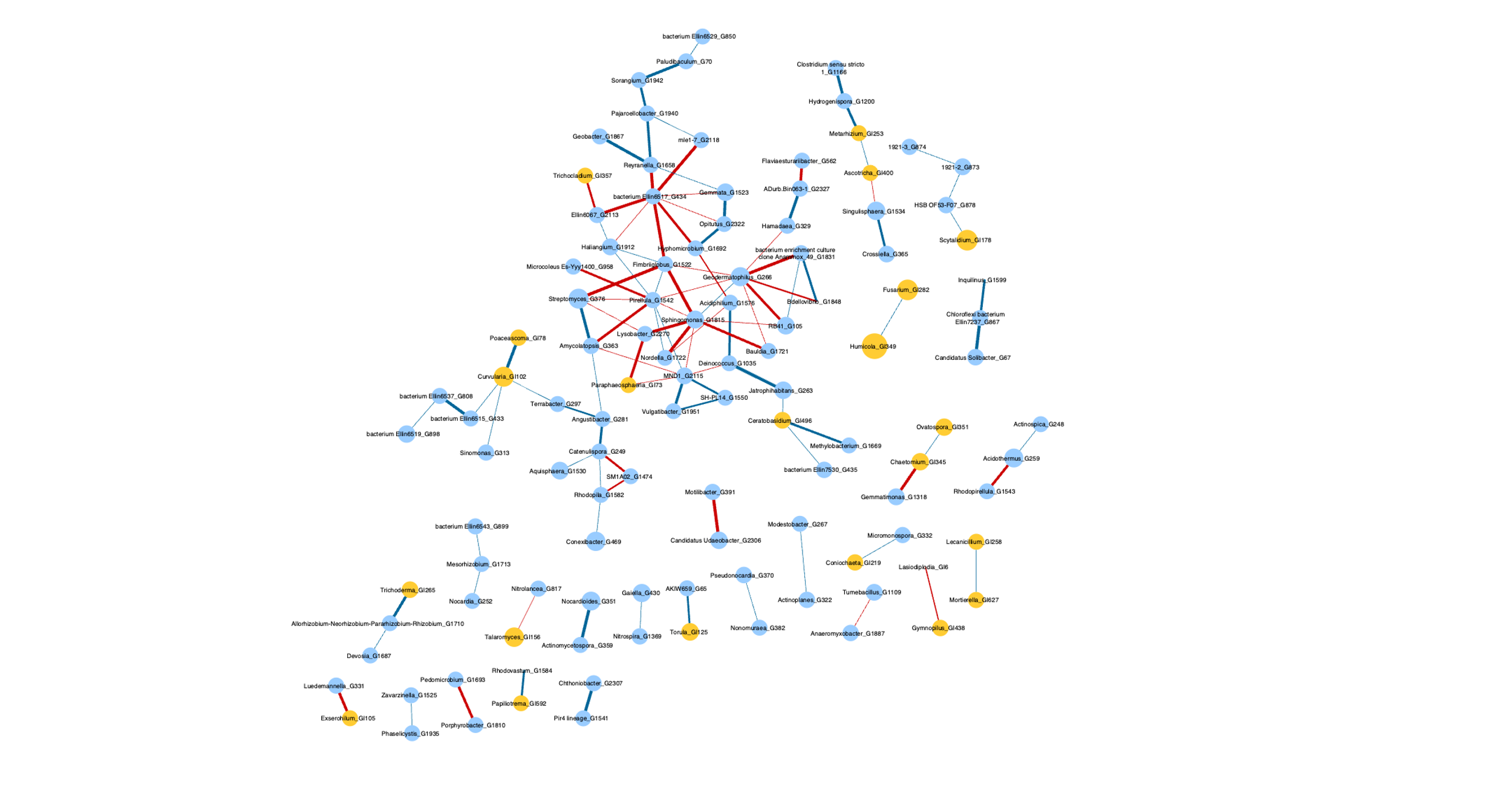


**C**


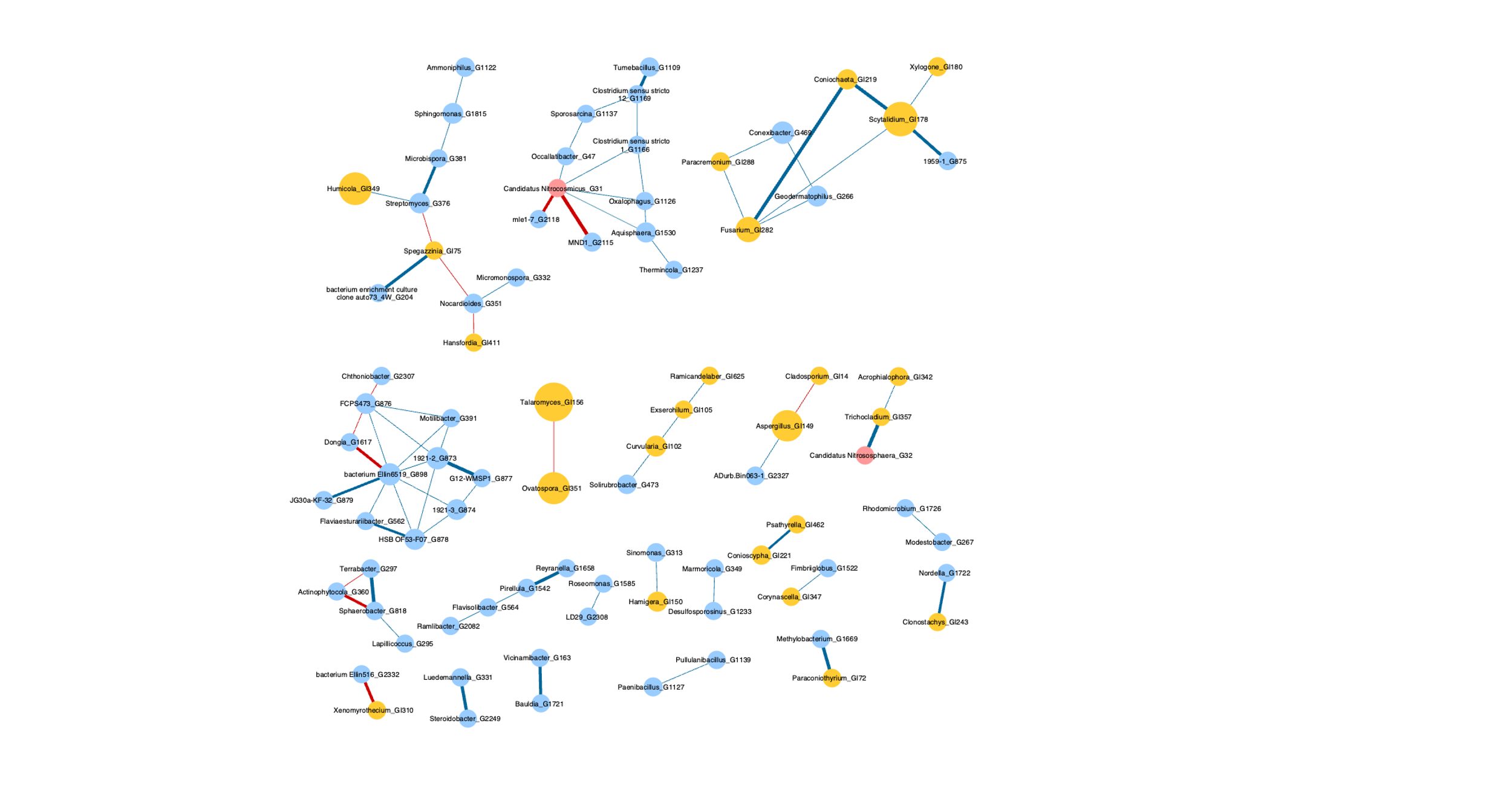


**D**

**S1 Fig**. Co-occurrence networks including (A) N-BAF, (B) N-BAT, (C) W-BAF, and (D) W-BAT. Node color represents domain (pink: archaea, blue: bacteria, and yellow: fungi). Size of the node is the average relative abundance based on three growth stages (TP, GP, and RP). Blue and red edges mean positive and negative correlations, respectively. Intensity of edge color represents a range of correlations.
