# Supplementary material for: Soil microbiome analysis reveals effects of periodic waterlogging stress on sugarcane growth
Source: PLoS One. 2023 Nov 2;18(11):e0293834. doi: 10.1371/journal.pone.0293834 (PMC10621937; doi:10.1371/journal.pone.0293834)
Supplement: Attachment — Submitted filename: Response to Reviewers.docx [file pone.0293834.s010.docx]

- **Response to the Reviewer #1**

**Reviewer #1**:

1. The draft titled “Soil Microbiome Analysis Reveals Effects of Period Waterlogging Stress on Sugarcane Growth”. The idea of experimental design is novel, and the work is meaningful. However, as a journal article to be published, there are many deficiencies.

**ANS:**

The title should be revised from “Soil Microbiome Analysis Reveals Effects of Period Waterlogging Stress on Sugarcane Growth” to “Soil Microbiome Analysis Reveals Effects of Periodic Waterlogging Stress on Sugarcane Growth”.

2. Results: The results can be further simplify.

The figures look uncomfortable, especially figure 5 about microbial network suggest visualization by cytoscape or gephi.

**ANS:**
We adjusted the results and the networks had already been visualized using Cytoscape. We adjusted the colors of the nodes and edges to increase contrast and added the node labels.

3. And the figure 1 should reflecting the abundance of archeobacteria.

**ANS:**

The 16S rRNA gene has the capability to detect both bacteria and archaea. Therefore, our taxonomic abundance analysis considered these two groups collectively. In presenting our results, we aim to underscore the leading ten phyla identified through both the 16S and ITS analyses. Since archaea constituted a minority within the community, they were categorized under 'Others'. Furthermore, other results were analyzed separately based on 16S rRNA and ITS genes.

4. As for Figures 2 and 3, it is also recommended to include analysis of archeobacteria.

**ANS:**

From the previous answer, we analyzed separately based on 16S rRNA and ITS genes. So, the archaeal group was included in both diversity analyses.

5. Discussions: This should have a thorough discussion with the results of previous studies, not showcasing the results.

**ANS:**

We have adjusted the discussion to be more comprehensive and not just a showcase of the results.

- **Response to the Reviewer #2**

**Reviewer #2**:

The overall structure of the article is complete and the content is substantial, but there are still some small problems that need to be added.

1. Line 24: The abstract need to be further condensed.

**ANS:**

The abstract was condensed as per your suggestion.

2. Line 39-41: These contents are not the results of this paper.

**ANS:**

We have removed the suggested sentences.

3. Line 52: The Latin name for sugarcane needs to be supplemented, please refer to the study of Xiao et al. (2023) (<https://doi.org/10.1016/j.catena.2023.107000>) and Xiao et al. (2023) (<https://doi.org/10.1016/j.eti.2023.103244>)

**ANS:**

We have added the Latin name for sugarcane as *Saccharum officinarum* L.

4. Line 87: Other studies or results of periodic waterlogging on sugarcane plants or soils should be mentioned.

**ANS:**

We added more details about the effect of waterlogging on sugarcane growth and soil in the introduction part as in lines 73-83.

5. Line 104: The GPS coordinates (eg. latitude and longitude) of the test site, soil type and state, altitude and other relevant information about the test site need to be supplemented.

**ANS:**

We have included additional details about the test site in 'The Description of Sugarcane Field Sites' in the 'Materials and Methods' section, specifically in lines 94-99.

6. Line 107-111: Sugarcane planting information, fertilization, irrigation and field management information all need to be supplemented, please refer to the study of Yang et al. (2023) (<https://doi.org/10.1016/j.apsoil.2022.104699>)

**ANS:**

We added more details of fertilization and irrigation information in 'The Description of Sugarcane Field Sites' in the 'Materials and Methods' section, specifically in lines 99-106.

7. Line 151: Raw data from 16s and ITS sequencing should be uploaded to relevant databases such as National Center for Biotechnology Information (NCBI).

**ANS:**

16S rRNA and ITS gene sequence data associated with this article have been deposited at NCBI under BioProject accession number: PRJNA658446 and SRA accession numbers: <SRR14677846> - [SRR14679243](https://trace.ncbi.nlm.nih.gov/Traces/sra?run=SRR14679243).

8. Line 162-164: As far as we know, the Shannon and Simpson indices are used to indicate microbial diversity, why not analyze microbial abundance (generally indicated by Chao1 and Ace indices)?

**ANS:**

We additionally analyzed the Chao1 index and inserted it in Fig 2.

9. Line 164: According to the format requirements of the journal, “p” should be rewritten as “P”.

**ANS:**

We have revised it.

10. Line 190-191: This sentence should be moved to the materials and methods section.

**ANS:**

We have moved it to the Materials and Methods section.

11. Line 192-194: Has the data in Table 2 been analyzed for significance? Why the relevant marks and descriptions are not showed in Table 2 and table notes?

**ANS:**

Yes, we have additionally analyzed the significance of sugarcane characteristics between normal and periodic waterlogged fields and provided raw data on sugarcane characteristics in S2 Table.

12. Line 206: Do these soil chemical properties differ significantly between the normal field and the periodic waterlogged field?

**ANS:**

We collected soil samples from five points for each field and then pooled them together to measure the soil's chemical properties. Unfortunately, we could not compare it using any statistical test because we did not have replicates for each measurement. Thus, we could not really say whether soil chemical properties between normal and periodic waterlogged fields differ significantly or not.
