# Supplementary material for: Soil microbiome analysis reveals effects of periodic waterlogging stress on sugarcane growth
Source: PLoS One. 2023 Nov 2;18(11):e0293834. doi: 10.1371/journal.pone.0293834 (PMC10621937; doi:10.1371/journal.pone.0293834)
Supplement: Attachment — Submitted filename: Response to Reviewers.docx [file pone.0293834.s011.docx]

**Response to reviewers**

Dear editor and Reviewers

We have edited the manuscript as per your suggestions, and we have reviewed the references included in the manuscript.

Comment: Lines 116-120: How are soils treated and preserved for soil chemical property analysis? For example, what is the sieve size in millimeters?

Answer: We have included details on how soils are treated and preserved for soil chemical property analysis in the Materials and Methods section, specifically in the section on Soil Chemical Property Analysis, as per your suggestion (lines 133-139).

Comment: Lines 236-238: References need to be provided.

Answer: We have already added a reference as per your suggestion, and it can be found in line 234.

Comment: Lines 273, 326, 345: "p" may be rewritten as "P."

Answer: We have made the change to capitalize "p" as per your suggestion.

Comment: Line 285: "shows" may be rewritten as "showed."

Answer: We have made the change to use "showed" as you recommended.

Comment: Line 425: "...[80-82]. Importantly, ..."

Answer: We have revised as you suggested.

Sincerely,

Jiraporn Jirakkakul
